# Barriers to Rural Induced Abortion Services in Canada: Findings of the British Columbia Abortion Providers Survey (BCAPS)

**DOI:** 10.1371/journal.pone.0067023

**Published:** 2013-06-28

**Authors:** Wendy V. Norman, Judith A. Soon, Nanamma Maughn, Jennifer Dressler

**Affiliations:** 1 Department of Family Practice, Faculty of Medicine, University of British Columbia, Vancouver, Canada; 2 Women’s Health Research Institute, British Columbia Women’s Hospital, Provincial Health Services Authority, Vancouver, Canada; 3 Faculty of Pharmaceutical Sciences, University of British Columbia, Vancouver, Canada; Indiana University, United States of America

## Abstract

**Background:**

Rural induced abortion service has declined in Canada. Factors influencing abortion provision by rural physicians are unknown. This study assessed distribution, practice, and experiences among rural compared to urban abortion providers in the Canadian province of British Columbia (BC).

**Methods:**

We used mixed methods to assess physicians on the BC registry of abortion providers. In 2011 we distributed a previously-published questionnaire and conducted semi-structured interviews.

**Results:**

Surveys were returned by 39/46 (85%) of BC abortion providers. Half were family physicians, within both rural and urban cohorts. One-quarter (17/67) of rural hospitals offer abortion service. Medical abortions comprised 14.7% of total reported abortions. The three largest urban areas reported 90% of all abortions, although only 57% of reproductive age women reside in the associated health authority regions. Each rural physician provided on average 76 (SD 52) abortions annually, including 35 (SD 30) medical abortions. Rural physicians provided surgical abortions in operating rooms, often using general anaesthesia, while urban physicians provided the same services primarily in ambulatory settings using local anaesthesia. Rural providers reported health system barriers, particularly relating to operating room logistics. Urban providers reported occasional anonymous harassment and violence.

**Conclusions:**

Medical abortions represented 15% of all BC abortions, a larger proportion than previously reported (under 4%) for Canada. Rural physicians describe addressable barriers to service provision that may explain the declining accessibility of rural abortion services. Moving rural surgical abortions out of operating rooms and into local ambulatory care settings has the potential to improve care and costs, while reducing logistical challenges facing rural physicians.

## Introduction

Induced abortion is a common procedure with 95,876 reported in Canada, [1] and 43.8 million globally, [2]for 2008. Nearly a third (31%) of Canadian women have at least one induced abortion during their reproductive lifespan [3]. We define induced abortion, and will use the term “abortion”, with the Canadian Institute for Health Information definition: “Induced abortion is defined as the medical termination of pregnancy” [1]. Availability of abortion service in rural areas is a growing problem in Canada [4]–[8]. Abortion is increasingly available only at purpose-specific clinics (“abortion clinics”). In Canada abortion clinics are located exclusively in the largest urban centers [4]–[8]. Statistics Canada reports a steady downward trend from 91% of abortions performed in hospitals in 1988 (when the provision of abortions at non-hospital clinics became legally available in Canada) to 43% in 2010 [1], [9], [10]. Due to the absence of abortion clinics in rural areas of Canada, surgical abortion in rural areas is available only within hospitals. As such, and given that the overall number of abortions performed has been stable over this interval, [1], [9], [10] the decline in the proportion of all abortions that are performed in hospitals represents at least a 58% decline in the *number* of abortions performed in rural areas.

British Columbia (BC) is a large Canadian province of about 4 million people, with an area aproximately the size of France and Germany combined. BC maintains a comprehensive provincial referral system for abortions, the Pregnancy Options Service (POS) [11]. The POS system offers a unique opportunity to study factors influencing abortion availability and distribution. POS records indicate that the number of rural abortion providers, as well as the number of communities where abortion service is offered, have declined more than 60% over the decade prior to 2010 [12]. With declining rural access to surgical abortion, abortion induced using medication (“medical abortion”) could be an available alternative. However several sources currently estimate medical abortions are fewer than 4% of all Canadian abortions [6], [10], [13].

No published studies have examined experiences and practice among rural Canadian physicians providing abortion. In Canada only physicians are licensed to provide abortions. Experiences and practice among urban abortion providers who are voluntary members of a professional association in Canada, the USA and Australia have been previously reported [14]–[16]. These studies demonstrate adherence to evidence-based guidelines and a large degree of uniformity of practice among urban abortion providers in all three jurisdictions. All BC urban abortion clinics were members of this association in 2001 and participated in the most recently published survey: none of the rural abortion providers in BC participated in the previous surveys.

Thus, rural abortion services are disappearing in Canada although we know little about the experiences of abortion providers and factors related to their provision of rural services. Understanding the distribution, services provided and conditions experienced by rural abortion provider physicians is a first step to determine appropriate health policy. Determination of factors that could address the remarkable shift away from abortion services in rural areas has the potential to improve closer-to-home access to reproductive health care for rural and remote Canadians.

## Methods

### Ethics

Ethics board approval was obtained from the University of British Columbia Children’s and Women’s Hospital Research Ethics Board (H11-00766) prior to commencement of the study. All participants submitted written completed surveys, including a statement that submission of the survey would imply consent to participation. Participants had the option to provide self-identification at the beginning of the survey, with the assurance that no data would be published that would allow individual identification.

This mixed methods study explored practices and experiences of rural compared to urban abortion providers in BC. Self-administered questionnaires were distributed to BC abortion providers in 2011. Questionnaire respondents were invited to participate in a semi-structured individual interview. (See qualitative findings in the companion article: “The perspective of rural physicians providing abortion in Canada: qualitative findings of the BC Abortion Providers Survey (BCAPS)”).

### Questionnaire

The questionnaire was kindly provided by Drs. Lichtenberg, O’Connell White, Paul and Jones as developed for their previously published studies [14]–[16]. We adapted their instrument to remove questions or options inappropriate for the Canadian context or the purpose of this study, and to add options pertinent to Canada, creating the “BC Abortion Providers Survey” (BCAPS). Respondents selected among coded responses for all questions, and were encouraged to write-in comments. This report presents the analysis of abortion provider demographics and distribution; basic services; and reported stigma or harassment. The companion article in this issue: “*The perspective of rural physicians providing abortion in Canada: qualitative findings of the BC Abortion Providers Survey (BCAPS)*”, explores qualitative findings on attitudes, perceived barriers and facilitators related to abortion provision.

### Sampling Technique

We distributed the BCAPS between April 15^th^ and September 15^th^, 2011 to all abortion providers on the POS roster. We defined “urban” participants as those providing services within a Census Metropolitan Area (CMA). Statistics Canada defines a CMA as being “formed by one or more adjacent municipalities centred on a large urban area (known as the urban core). The census population count of the urban core is at least 100,000 to form a census metropolitan area. To be included in the CMA, other adjacent municipalities must have a high degree of integration with the central urban area, as measured by commuting flows derived from census place of work data.” [17], [18] All other participants were defined as rural providers.

### Statistical Analysis

We analyzed questions referring to individuals using the individual clinician as the unit of analysis. We used the facility (either an urban abortion clinic, or a rural hospital or rural community as appropriate) as the unit of analysis for questions regarding facilities. In some cases it was appropriate to use the health authority region, i.e., the administrative division for delivery of health services within BC, which we will refer to as the “region”, [19] as the unit of analysis. Only physicians are licensed to provide abortion service in Canada, thus all responders are licensed physicians. We performed all analyses using the software program R [20]. Where appropriate, we used the students’ t-test (comparing age of providers), Chi-square test of independence (comparing gender and location, and years in practice and location), Fischer’s exact test (comparing specialty mix and location)and a Wilcoxon rank sum test (comparing proportion of practice devoted to family planning).

## Results

**Figure 1:**
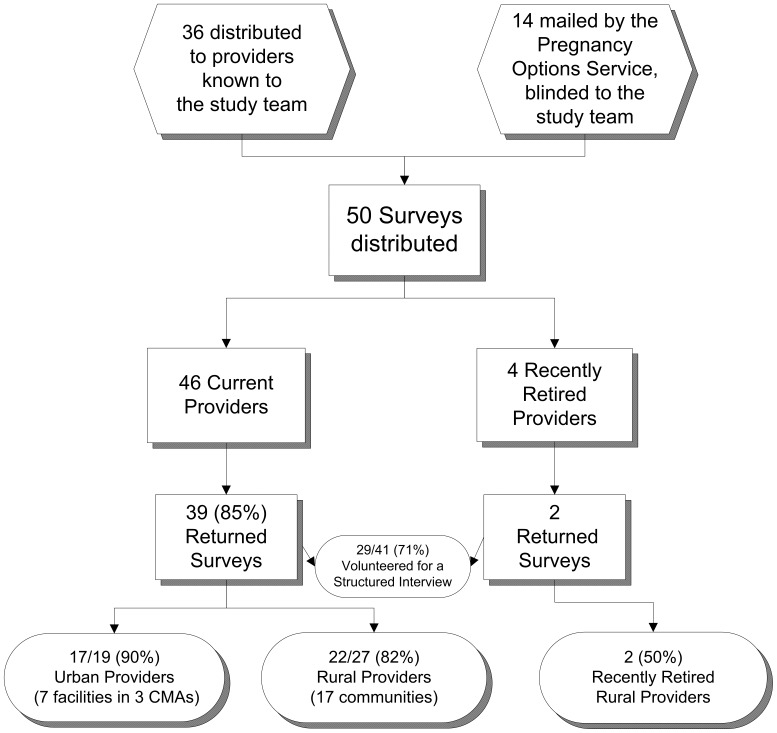
Survey Distribution and Response. Notes: CMA = Census Metropolitan Area.

Eighty-five percent (39/46) of current surgical abortion providers in BC returned surveys.(Fig. 1) Two additional rural physicians returned surveys. Although each of these had formerly provided abortion service and were still working as physicians in their communities, both had recently discontinued providing abortions. This analysis presents responses from current providers for all services questions, and considers both current and past providers in presentation of questions regarding harassment and stigma.

### Demographics of Abortion Providers

Rural providers were more likely to have been providing abortions for less than 15 years (12/22, 54.5%), although of a similar average age, and proportion of oldest physicians, than urban providers (3/17, 17.6%, p = 0.044) (Table 1).

**Table 1:** Characteristics of rural compared to urban current abortion provider participants.

	Overalln = 39	Rural Providersn = 22	Urban Providersn = 17	Significance(rural vs. urban)
**Average Age (SD) in years**	51.8 (10.7)	50.7 (10.7)	53.1 (11.1)	p = 0.50
Current age over 59 years	11 (28%)	6 (27%)	5 (29%)	
**Female Providers**	21 (54%)	11 (50%)	10(59%)	p = 0.82
**Specialty (%)**				p = 0.58
Obstetrician-gynecologist^1^	21 (54%)	12 (55%)	9 (53%)	
Family Physician^2^	18 (46%)	10 (45%)	8 (47%)	
**Years as an abortion provider**				
<15 years	15 (38%)	12(55%)*	3 (18%)*	p = 0.04^3^
>15–25 years	12 (31%)	5 (23%)	7 (41%)	
>25 years	12 (31%)	5 (23%)	7 (41%)	
**% Practice in Family Planning**	30.7(SD30.1)	15.0(SD11.2)*	51.0 (SD35.9)*	p<0.001

Notes:

* Significant differences.

1 Including those with Canadian and Non-Canadian certification.

2 Including general practitioners.

3 Siginificant when Rural vs Urban for <15 years is compared to 15 or more years.

Respondents indicated an overall average of 30.7% (SD 30.1%) of their practice was devoted to family planning, although 5/17 (29.4%) urban providers reported that they practice exclusively in this area. Among urban providers, 7/17 (41%) indicated family planning as 30% or less of their practice. Among rural providers 21/22 (95%) indicated family planning as 30% or less of their practice, and none practice exclusively in family planning.

### Number of Abortions

Responding providers reported an estimated 15,953 induced abortions performed in 2010 in BC, including 2349 (14.7%) medical abortions. The average number of reported abortions performed by each rural provider was 76 (SD 52) including 35 (SD 30) medical abortions. The urban facilities provided 90.2% of all reported abortions by any method.

### Distribution of Abortion Services

Urban providers work within seven facilities: six abortion clinics (15/17, 88.3%), and one urban hospital (2/17, 11.8%). Rural providers identified 16 distinct communities in which they practice. One rural provider identified only the region of practice. For analysis purposes this provider was assumed to represent a distinct 17^th^ community, an assumption supported by the variation in facility and service descriptors between this responder and those of other responders in the same region.

Abortion services were reported in four of the five geographic health authority regions. (Table 2) No sources available were able to identify provision of induced abortion in one large region, including in the encompassed CMA. Three quarters of rural communities (13/17, 76.5%) do not offer induced abortion beyond the first trimester (Table 3). Only 1.7% of second trimester surgical abortions reported occurred in rural communities. By contrast, medical abortions in the first trimester of pregnancy are relatively accessible with 12/17 (70.6%) of reporting rural communities offering this service compared to 2/7 (28.6%) urban facilities.

**Table 2:** Location of facilities where participants provide surgical abortion services.

Health Authority Region	% of BC females age 15–44^1^	Rural^2^n = 17	Urban^3^n = 7	Totaln = 24
Provincial Health Services^4^		n/a	1	1
Fraser Health	36.5%	0	0	0
Interior Health	14.1%	4	1	5
Northern Health	6.3%	7	n/a^5^	7
Vancouver Coastal Health	28.1%	1	4	5
Vancouver Island Health	15.1%	5	1	6

Notes:

1 BCStats. Population Estimates by Standard Age Groups. [37].

2 The unit of reporting is by rural community which in this case is equivalent to facility.

3 The unit of reporting is by urban facility.

4 Provincial Health Services Authority (PHSA) facilities are located in Vancouver and have a provincial service mandate. “(The PHSA’s) primary role is to ensure that BC residents have access to a coordinated network of high-quality specialized health care services.” [38].

5 Northern Health Authority does not have a Census Metropolitan Area (see definition of urban in text); Fraser, Interior, Vancouver-Coastal and Vancouver-Island each have one.

**Table 3:** Rural compared to urban induced abortion service availability reported for BC.

Service	Rural^1^n (%)	Urban^2^n (%)
**Surgical Induced Abortion**	17 (100)	7 (100)
Upper limit of gestational age (weeks)		
12 or less	8 (47.1)	1 (14.3)
>12 up to 14	5 (29.4)	2 (28.6)
>14 up to 18	4 (23.5)	1 (14.3)
Over 18	0	3 (42.9)
**Medical Abortion (First trimester)**	12 (70.6)	2 (28.6)
**Medical Abortion (Second trimester)**	10^3^ (58.8)	2 (28.6)

Notes:

1 The unit of reporting is by rural community.

2 The unit of reporting is by urban facility.

3 Although not specifically elicited, about half of rural facilities volunteered information on restricted criteria for medically induced second trimester abortions, such as restriction to fetal indications.

Knowledge of the location and the mode of anaesthesia available for the provision of surgical abortion are relevant in order to understand the barriers and facilitators experienced by providers. All rural providers performed surgical abortions within a hospital operating room, although three indicated availability of hospital outpatient clinic space where they performed a portion of their cases. Among urban respondents, four of the seven facilities performed some or all procedures within a hospital, but only one exclusively used an operating room, with the others predominantly using outpatient based clinic space. Two of these clinics also offered procedures within an operating room for exceptional indications.

With respect to the anaesthesia and analgesia methods offered by facilities providing surgical abortions: all urban clinics (7/7, 100%), and 6/17 (35.3%) rural facilities, offered local anaesthesia plus intravenous sedation as the predominant modality for anaesthesia. This approach was utilized for over 90% of surgical abortions. Examination of physician practice indicates that among rural providers 16/22 (72.7%) offer general anaesthesia as an option and 10/22 (45.5%) use general anaesthesia exclusively or nearly exclusively.

### Stigma, Harassment and Logistical Barriers

Nearly half (8/17, 47.1%) of rural communities reported a proportion of operating room nurses or anaesthesiologists who would not accept an assignment to the abortion cases. Operating room scheduling issues were noted as a significant source of stress and/or conflict by rural abortion provider respondents, nearly all of whom wrote-in extensive additional notes. For example: “I have suffered threats and have had both (*sic*) anesthetists, [ultrasound] technologists, and [operating room] nurses refuse to cooperate in treatment or have had patients suffer insults”. No such barriers were reported from providers in the urban facilities who conversely reported supportive facility environments. In addition, 7/22 (31.8%) of rural providers indicated their community had experienced resignations from physicians or nurses due to unwillingness to endure harassment or stigma experienced as a result of involvement in abortion provision. No such resignations were reported from urban facilities or providers. The only reports of sporadic anonymous personal harassment or violence were among urban providers, and were in the categories: “threats to you or your family” (2/17, 11.8%); “property vandalism”(4/17, 23.5%); and “trespassers at your home”(2/17, 11.8%).

We asked all respondents to consider 12 possible categories of relationships (e.g., siblings, parents, children, colleagues, and friends) and report which were aware of their work as an abortion provider. No differences were seen between rural and urban physicians overall or among any specific categories.

## Discussion

BC’s current abortion providers are about half specialists and half family physicians, and about half are female, both overall and in each jurisdiction. Rural abortion service was reported as available in 17 hospitals outside of large urban areas in BC. Urban service was reported as available at seven facilities in three of the four largest urban areas. Our finding that 15% of all abortions are medical abortions is higher than previously reported in Canada. Rural physicians perform an average of 76 (SD 52) abortions annually, about half of which are medical abortions. Rural physicians report stigma and operational barriers within their facility and among their colleagues with particular conflict arising in operating room scheduling. Urban physicians report excellent support from their facilities and colleagues, but occasional experiences of anonymous harassment and violence.

### Demographics of Abortion Providers

The mean age of BC abortion providers at 51.8 (SD 10.7) years is not significantly different than that reported for physicians overall in BC at 49.5 years (p = 0.50), with an equivalent proportion (28%) over age 60 [21]. A significantly greater proportion of rural compared to urban providers have been providing abortion service for less than 15 years. Data on the number of years in practice were not collected, thus we were unable to determine if the fewer years providing abortions among rural physicians represented attrition after a certain number of years of rural abortion service provision, or that rural physicians began providing abortions later in their careers, or was due to some other etiology. Further study of this phenomenon is warranted.

### Number of Abortions Performed in BC

The report of 15,953 induced abortions in 2010, is comparable to Canadian Institutes of Health Information (CIHI) report of 12,149 in the same year, as CIHI notes data from BC clinics to be incomplete [10]. The overall number of abortions is consistent with that reported by CIHI for BC in 2007 (15,770) and for 2011 (14,341), years for which complete data are reported [22], [23]. Urban case numbers reported per facility are thought to be accurate, however, rural physician self-reported ‘number of cases’ did exhibit rounding in some cases. As rural physicians contributed less than 10% of total BC abortions, the impact of rounding among individual physicians on the total number of reported abortions is likely to be minimal. Our respondents reported providing medical abortions as 15% of all abortions. This is significantly higher than analyses of administrative databases, which estimate fewer than 4% of abortions in Canada are medical abortions [6], [10], [13]. As medical abortion provision is difficult to capture through administrative database analysis for a number of reasons, our findings may represent a more accurate reflection of the provision of medical abortion in Canada.

### Distribution of Abortion Services

We found a mismatch between where abortion service is available, and where reproductive age women reside in BC. Over 90% of abortions reported for BC are offered in three of the four large urban areas(CMAs) in this province although only 57% of BC reproductive age women live in the regions associated with these areas [24], [25]. No urban or rural abortion services were detected within one regional health authority, although this region provides health care for 36.5% of all reproductive age women in BC [26], [27]. Abortion service in BC is provided in only 17/67 (25.4%) hospitals [28] outside of the large urban areas. A 2006 analysis [8] found 29/90 (32%) of all BC hospitals (i.e., both urban and rural) offered abortion service. However, four years later we have found abortion service offered at only 21/97 (22%) of all hospitals in BC. Both figures include abortion services at four urban hospitals. Adjusting for urban hospital-based services, this translates to a one-third decline (from 25 to 17 hospitals) in rural abortion service over 4 years.

### Stigma, Harassment and Logistical Barriers

Physicians providing abortions in rural areas are more likely than their urban counterparts to perform abortions in an operating room (100%), and to use general anaesthesia (73% include this method as an option while overall 46% use this method always). Rural providers, including responses from two former abortion providers who continue as practicing physicians in their community, report stigma and operational barriers within professional relationships and at their hospitals. This may suggest a role for frustration and early burn-out as an etiology for the previously noted marked rural abortion provider attrition. (See companion article “The perspective of rural physicians providing abortion in Canada: qualitative findings of the BC Abortion Providers Survey (BCAPS)” for related experiential findings from rural abortion providers.).

Although rural providers report prevalent use of general anaesthesia for abortion care, guidelines of expert organizations in Canada and globally [29]–[32] currently suggest local anaesthesia with or without sedation as preferred over general anaesthesia.

We found rural abortions are provided predominantly in hospital operating room settings, which physicians identified as a factor in both limitation to services and contributing to their experience of conflict, harassment and stigma. Urban hospital-based abortions are provided predominantly in non-operating room settings, similar to those for out-patient procedures such as colposcopy or colonoscopy. Health system advantages such as cost-effectiveness (for surgical abortion management without general anaesthesia), and a reduction in post-operative complications among services delivered in ambulatory care settings, have been documented for Canada and similar countries. [33]–[36].

### Limitations

The largest limitation to our understanding of the decline in rural physicians offering abortion service in BC is the inability to identify and sample most of the rural physicians who are no longer providing abortions. To partially address this, we plan to iteratively follow current and new providers over time to better understand challenges and facilitators. As POS has found providers offering only medical, and not surgical, abortion are less likely to participate in their register, we were unable to locate and survey all providers of medical abortions. Thus the number and proportion of actual medical abortions in the province may be higher than we report. POS uses a number of methods to ensure accurate information on current surgical abortion providers in the province of BC, aided by their role to distribute appropriate security updates and support for BC abortion providers on behalf of BC Women’s Hospital and the BC Ministry of Health. It is thought the POS register of surgical abortion providers is comprehensive. Self-reported estimates of procedures by rural physicians, although a small contribution to the total number of abortions in the province, may not correlate with the exact number of abortions performed. In contrast, the number of abortions provided by urban facilities is thought to be accurate.

### Conclusion

Attrition of rural abortion providers is an important problem throughout Canada. This study offers a first look at possible etiologies and potentially addressable issues. Rural induced abortion services in BC have limited accessibility, with sub-optimal alignment between where reproductive age women live and where services are available. Rural abortion service is nearly exclusively operating room based and usually under general anaesthesia, despite national and international recommendations for safe provision using local anaesthesia. Most rural abortion providers identified addressable barriers to service provision, in particular highlighting both stigma and service delivery conflicts where abortion service is limited to the hospital operating room. Moving surgical abortions out of operating rooms and into local ambulatory care facilities has the potential to lower costs while improving service availability in rural areas.
